# A script‐based method for achieving distortion‐free selected area electron diffraction

**DOI:** 10.1002/jemt.24124

**Published:** 2022-04-09

**Authors:** David R. G. Mitchell

**Affiliations:** ^1^ Electron Microscopy Centre, AIIM Building, Innovation Campus University of Wollongong Fairy Meadow New South Wales Australia

**Keywords:** DigitalMicrograph script, elliptical distortion, selected area electron diffraction

## Abstract

Electron diffraction patterns obtained on a TEM contain elliptical distortion resulting from column defects. This distortion can be corrected by applying offsets to the objective lens stigmators to cancel distortions occurring further down the column. In this work, a DigitalMicrographTM script‐based method has been developed to identify the optimum objective stigmator settings which produce a distortion minimum in diffraction. Initially, a manual (by eye) correction is used to determine the stigmator values necessary to bring the pattern distortion below the threshold at which it is no longer visible to the naked eye (<1%). Thereafter, an automated acquisition script is used to acquire matrices of diffraction patterns while varying the stigmator values about the values which were identified as producing a distortion minimum in the preceding step. This analysis can be applied iteratively to refine the location of the distortion minimum, using progressively finer step changes in objective stigmator values. The optimum stigmator values producing the distortion minimum in diffraction are very different to those in imaging. These imaging and diffraction stigmator values can be saved to script and subsequently recalled at the click of a button, making their application very simple. Using this method, diffraction pattern elliptical distortion in a newly installed TEM was reduced from 1.6% to 0.3%, on a measurement precision of 0.3%, effectively producing distortion‐free diffraction. The relevant scripts can be freely downloaded from the internet.

**Research Highlights:**

This paper reports a DigitalMicrograph script‐based method to identify and subsequently apply optimized objective stigmator values in diffraction mode. These effectively eliminate elliptical distortion inherent to this diffraction technique.

## Graphical abstract

- Elliptical distortion minimum obtained from an 11 × 11 matrix of diffraction patterns as a function of *x* and *y* objective stigmator settings.
- Recursive refinement of the distortion minimum, using increasingly finer stigmator step sizes, results in a five‐fold reduction of distortion.

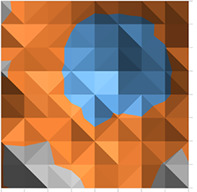

## 1 INTRODUCTION

In terms of precision and accuracy, electron diffraction in a transmission electron microscope (TEM) is a poor relation of neutron and x‐ray methods. Where the technique does excel is in its spatial resolution, permitting diffraction information from individual grains or defects to be obtained. The lack of a fixed diffracting geometry within the microscope, means that pattern centers are not fixed and software methods for locating centers and measuring diffraction spot/ring spacings and angles are required (Mitchell, 2008a; Mitchell, 2008b; Mitchell & Van den Berg, 2016). Even with software intervention, it can be difficult to measure d‐spacings with better than 1% precision due to the presence of geometrical distortion within the pattern. Such distortions can arise from lens defects in the microscope column (Capitani et al., 2006; Hou & Li, 2008; Mugnaioli et al., 2009), as well as magnetization retained in the viewing chamber casting (private communication). Off‐axis camera systems may also introduce distortion. As such, the magnitude and direction of distortion can vary significantly, even between identical models of microscope. This distortion is generally accepted by microscope vendors and techniques or procedures for correcting it at source are typically not available to installation engineers or sophisticated users. Typical microscope installation specifications call for measured distortions to be ≤2%, and provided the values fall below this threshold, the system is deemed to be performing satisfactorily.

This makes it somewhat of a lottery as to whether an installed system will be suitable for distortion‐free diffraction. A distortion in the range 1%–2% results in diffraction rings where the ellipticity is evident to the naked eye. Only once it falls below 1% can it become difficult to observe without using accurate measurement techniques such as graphical methods (Capitani et al., 2006; Hou & Li, 2008; Mugnaioli et al., 2009) or ellipse fitting analysis (EFA) (Mitchell & Van den Berg, 2016).

The EFA method has been incorporated into a convenient script (http://www.dmscripting.com/efanalysis.html) running in DigtalMicrograph™ (Gatan Inc.), which permits rapid analysis of suitable polycrystalline ring diffraction patterns. Gold nanoparticles sputtered onto a thin carbon support film, produce a high contrast, high intensity ring diffraction pattern which is suitable for assessing the performance of the microscope. The analysis determines the ellipse(s) which provide the best fit to the diffraction ring(s). The magnitude of the elliptical distortion is readily determined with relatively high precision (±0.3%). The distortion direction and magnitude of the distortion is fixed and if the microscope optics compensate for the effects of pattern rotation with changing camera length, as many do, then the same distortion vector/magnitude is present in all patterns, regardless of camera length. Where pattern rotation changes with camera length, then a lookup table of distortion (magnitude and angle) vs camera length would be required. The measurement of distortion typically takes only a few minutes per camera length and is easily done.

The elliptical distortion in a pattern can be nulled by compressing the pattern along the major axis and expanding it along the minor axis, and such a tool is included with the EFA script for post‐acquisition correction. Removing the geometric distortion originating from the microscope, then permits any lattice distortion due to crystal effects to be determined (Xiong et al., 2018). Graphical methods have also been used to carry out post‐acquisition measurement and correction of distortions enabling accurate lattice parameter determination in minerals (Capitani et al., 2006; Mugnaioli et al., 2009).

While such corrections enhance the precision with which crystallographic analysis can be performed, collection of distorted data followed by correction is less than ideal. A far better option would be to eliminate the distortion at the microscope. Hou and Li (2008) noted that the objective lens stigmators can be used to apply a distortion field to the objective lens, in order to counter the effects of distortion arising further down the column. There are some practical difficulties with this method. In diffraction mode, very large offsets to the objective stigmator settings are necessary in order to cancel typical distortions. In contrast, distortion in imaging mode is exquisitely sensitive to minor changes in objective stigmator settings. If stigmator adjustments producing a minimum in distortion in diffraction mode were left in operation upon returning to imaging mode, a grotesquely distorted image would ensue. Independent (and accessible) memory registers for these stigmator settings are therefore required for both imaging and diffraction modes. However, these are typically not available. Hou and Li (Hou & Li, 2008) identified an approximate minimum in diffraction pattern distortion by manually varying the objective stigmators across a coarse matrix of values. This is not trivial because on modern microscopes the objective lens stigmator controls on the console are often deliberately deactivated in diffraction mode. Sophisticated users can edit and revise stigmator values by directly adjusting software settings, but this is hardly facile nor does it make for routine implementation.

DigitalMicrograph™ (Gatan Inc.), is widely used in TEM systems as the software front‐end to Gatan's range of cameras and spectrometers. It also facilitates scripting control of the microscope (Mitchell & Schaffer, 2005) and so provides an elegant solution to the issues raised above. Two scripts have been developed. One (Obj. Stig. Scanner) automates the acquisition of a matrix of diffraction patterns while varying the objective stigmator settings, and from which a distortion minimum can be identified. The second (SADP Distortion Corrector) enables the respective objective stigmator values producing minimum distortion in imaging and diffraction modes to be saved and readily applied at the click of a button. It also permits changing the stigmator values, as the console stigmator controls may be disabled in diffraction mode. The scripts are made freely available via the author's website (www.dmscripting.com).

## 2 MATERIALS AND METHODS

All experiments were conducted on a recently commissioned, current generation TEM operating at 200 kV. The microscope vendor details of the system have been excluded. The distortion effects reported herein will be common to all manufacturers and it is not the author's intention to suggest that distortion of this type is a flaw/characteristic unique to one vendor or product. The solution provided here will be universally applicable, provided an instance of DigitalMicrograph™ is connected to the microscope. If not, but alternative scriptable software is available, then users can implement a similar approach using the coding platform of their choice. The specimen used was a gold on carbon diffraction waffle grating specimen, consisting of nanocrystalline gold sputtered onto carbon. Standard imaging and diffraction conditions were used (largest condenser aperture [200um], spot size producing the highest intensity beam) and the column aligned following the manufacturer's recommendations. To standardize the acquisition conditions the objective lens was set to standard focus and specimen height was adjusted to focus the image. Following column alignment, a region containing only thin, amorphous carbon was imaged in order to manually correct the objective stigmation to eliminate any astigmatism from the image. The brightness control was turned clockwise from the crossover point, until the beam was the same diameter as the fluorescent screen to produce parallel illumination. In diffraction mode, a camera length of 600 mm was used throughout, being the camera length most commonly used for typical materials science specimens on this instrument. A 50um selected area aperture was used to select a region of gold nanoparticles. Switching to diffraction mode resulted in diffraction rings which were mostly continuous under these conditions. The column was degaussed twice to remove any lens hysteresis. The pattern was then fine focused with the Diffraction Focus control and the intermediate lens stigmators were adjusted to make the transmitted beam as round and sharp as possible. The success of this method and the accuracy of electron diffraction in general, hinge upon maintaining consistent diffraction conditions, especially a fixed objective lens (use Standard Focus) and a constant Intermediate Lens value (Diffraction Focus). Patterns (2 k × 2 k) were acquired on a Rio16 camera (Gatan Inc.), integrating for 8 s. Pattern distortion was measured using the EFA analysis script.

The microscope in this work was not fitted with an energy filter. Energy filters, either in‐column or post‐column, will introduce their own distortion. Only post‐column filters actively correct for this. Although not tested on energy filtered microscopes, the scripting approach reported here should work on such systems, provided the filter is operated under fixed conditions during calibration and use. However, unless energy filtering is specifically required, such microscopes are best avoided in preference for those offering direct capture to camera.

## 3 RESULTS AND DISCUSSION

Table 1 summarizes the various diffraction pattern distortion measurements made during the analysis. The as‐installed (uncorrected) elliptical distortion was 1.6%. This was within the installation specification (≤2%) but was of sufficient magnitude to be evident to the naked eye. In DigitalMicrograph™, a circular annotation was overlaid on one of the larger rings in a pattern derived from polycrystalline gold and the elliptical distortion was immediately apparent. The first stage of correction was to use a coarse (by eye) method to determine the objective stigmator values which would make the pattern approximately circular. This was done by having a live diffraction pattern updating on the screen with a circular annotation superimposed on one of the larger rings (the diameter being approximately 2/3rds the pattern width) and then coarsely adjusting the objective stigmators to make the pattern concentric with the circular annotation.

**TABLE 1:** Diffraction pattern distortion values measured after adjusting the objective stigmator values in various correction steps

Comment	Distortion/%	*X* obj. stig.	*Y* obj. stig.
As‐installed	1.61	34,656^a^	36,640^a^
Coarse (by eye) correction	0.78	30,656	40,640
Coarse matrix (step = 2500)	0.59	32,500	42,500
Fine matrix (step = 1000)	0.30	32,500	40,500

^a^ Optimum values for distortion‐free imaging.

**FIGURE 1:**
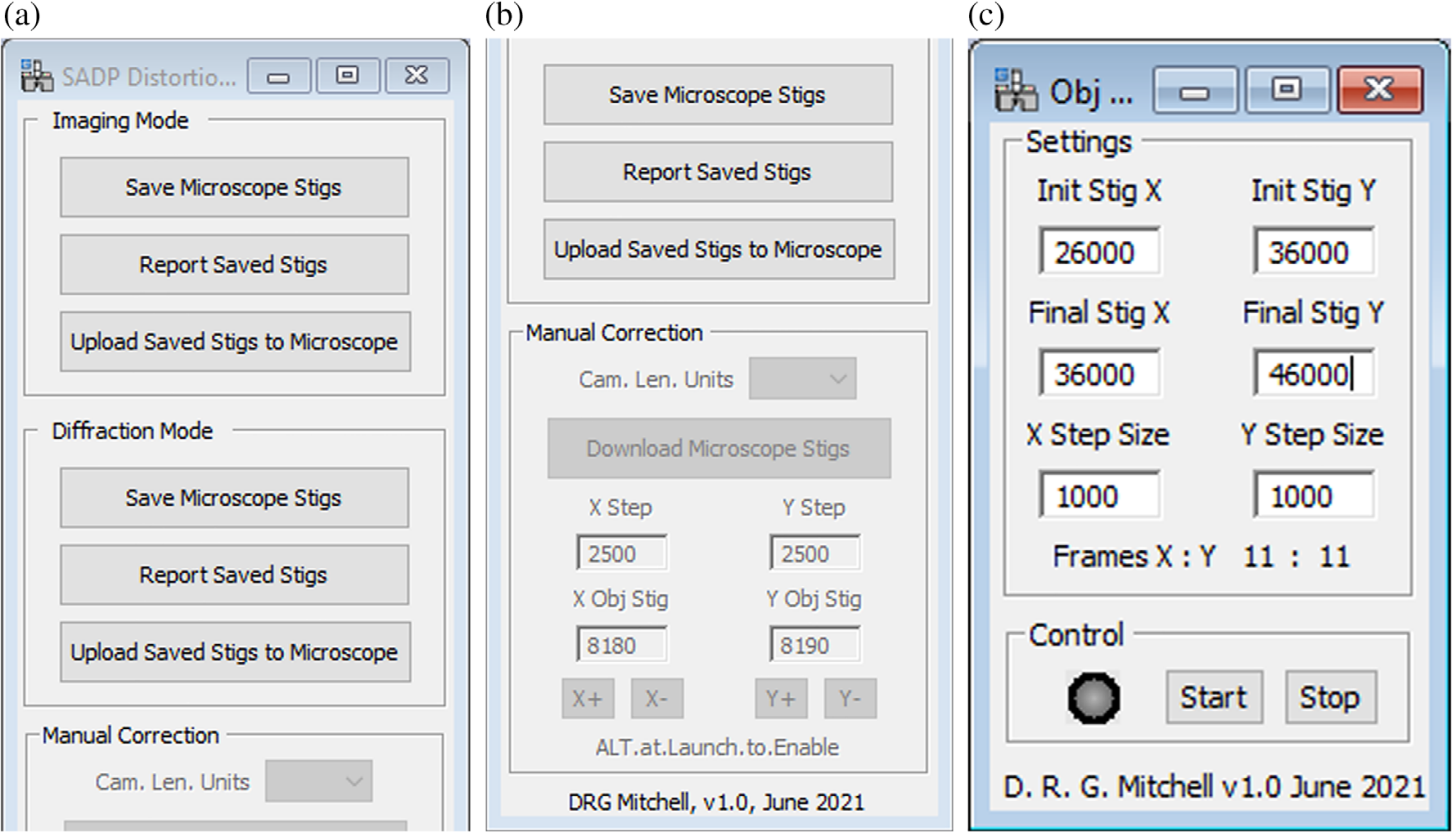
Scripts used in this work: (a) and (b) upper and lower sections of the SADP distortion corrector script, respectively. The upper section (a) provides memory registers for saving and recalling objective stigmator settings in imaging and diffraction modes, while the lower section (b) permits manual control of the objective stigmators; (c) Obj. Stig. Scanner script which automates the acquisition of a matrix of diffraction patterns while stepping through the range of objective stigmator values specified

The microscope disables the console objective stigmator controls in diffraction mode and so these could not be used for this adjustment. The SADP Distortion Corrector script (Figure 1a–b) was used for this correction. This has +/− buttons which allow step changes (of size specified in the *X*/*Y* Step field) to be applied to the objective stigmators. Large changes in objective stigmator values also cause the pattern to move slightly, so some adjustment of the projector lens alignment shift controls was needed to keep the transmitted beam centered on the beam stopper, along with some recentring of the circular annotation on the pattern. Once the live diffraction pattern appeared reasonably circular (distortion values of ≤1% are achievable with this method), a high quality (integrated) pattern was acquired and analyzed with the EFA script. This showed (Table 1) that the distortion had halved to 0.78%. The precision of the EFA method is around ±0.3%, which suggested that further refinement of this minimum should result in further improvement.

**FIGURE 2:**
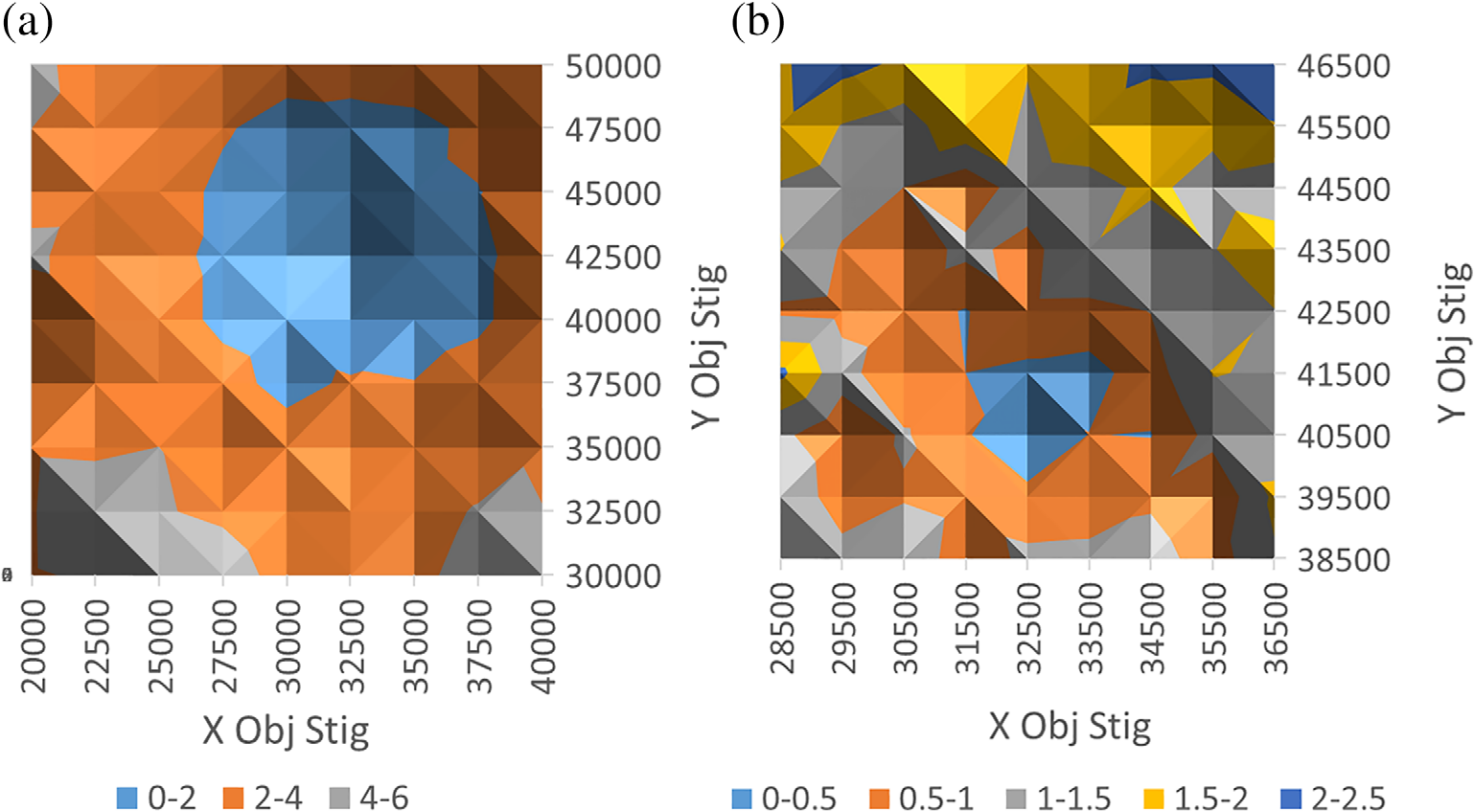
Diffraction pattern elliptical distortion as a function of *X* and *Y* objective stigmator settings, derived from 11 × 11 matrices of diffraction patterns captured using the Obj. Stig. Scanner script: (a) Coarse Matrix using an objective stigmator step size of 2500; (b) Fine Matrix, centered on the minimum in (a) and using an objective stigmator step size of 1000. The color legend shows the distortion values in %

The objective stigmator values determined by the coarse (by eye) method were then used as the central point about which an 11 × 11 matrix of diffraction patterns were acquired at various stigmator settings, using a coarse step change in objective stigmator *X* and *Y* values of 2500 (Coarse Matrix). Note, the range of possible stigmator values is 0–65,535. The Obj. Stig. Scanner script (Figure 1c) automates the acquisition process by stepping through a range of objective stigmator *X* and *Y* values while capturing and saving the diffraction patterns. The patterns were subsequently analyzed using the EFA script. Given the relatively high uncertainty (ca 0.3%) when measuring distortion values of just a few % or lower, the matrix of distortion values was plotted as a surface to identify the minimum visually, rather than simply selecting a single measurement yielding the lowest distortion value. Figure 2a shows the variation in distortion in the Coarse Matrix as a function of the *X* and *Y* objective stigmator values. The minimum in distortion was 0.59%, a decrease from 0.78% achieved using the coarse (by eye) method. In turn, the stigmator values producing this new minimum defined the center point for a second (Fine) Matrix, this time using a step size of 1000 (Figure 2b). The localization of the distortion minimum is less clear in the Fine Matrix (Figure 2b) than in the Coarse, due to the smaller range of measured distortion values relative to the uncertainty (0.3%). The measured distortion at the optimum objective stigmator values in the Fine Matrix was 0.3%. Since this is the same as the uncertainty in the measurement technique, further improvement is unlikely to be achieved by scanning at finer step sizes.

In order to apply the optimum objective stigmator values for imaging and diffraction modes, the SADP Distortion Corrector script was used (Figure 1a). In imaging mode, amorphous carbon was used to correct image distortion by manually adjusting the objective stigmators to produce an astigmatism‐free image in the usual manner. These stigmator values were then saved to the script by clicking on the Imaging Mode: Save Microscope Stigs button in the script. In practice, given the acute sensitivity of image astigmatism to the objective stigmator settings, the values saved for imaging would likely be updated at each diffraction session. The microscope was then switched to Diffraction mode and the optimum objective stigmator values of *X* and *Y*, determined from the Fine Matrix (Table 1), were entered in the Manual Correction: *X*/*Y* Obj Stig fields of the script (Figure 1b). These values are uploaded directly to the microscope as soon as they are entered. They were then saved to the script by clicking on the Diffraction Mode: Save Microscope Stigs button. Thereafter, these saved values could be uploaded from the script to the microscope by switching to the relevant mode (Imaging or Diffraction) and clicking on respective Upload Saved Stigs to Microscope button.

**FIGURE 3:**
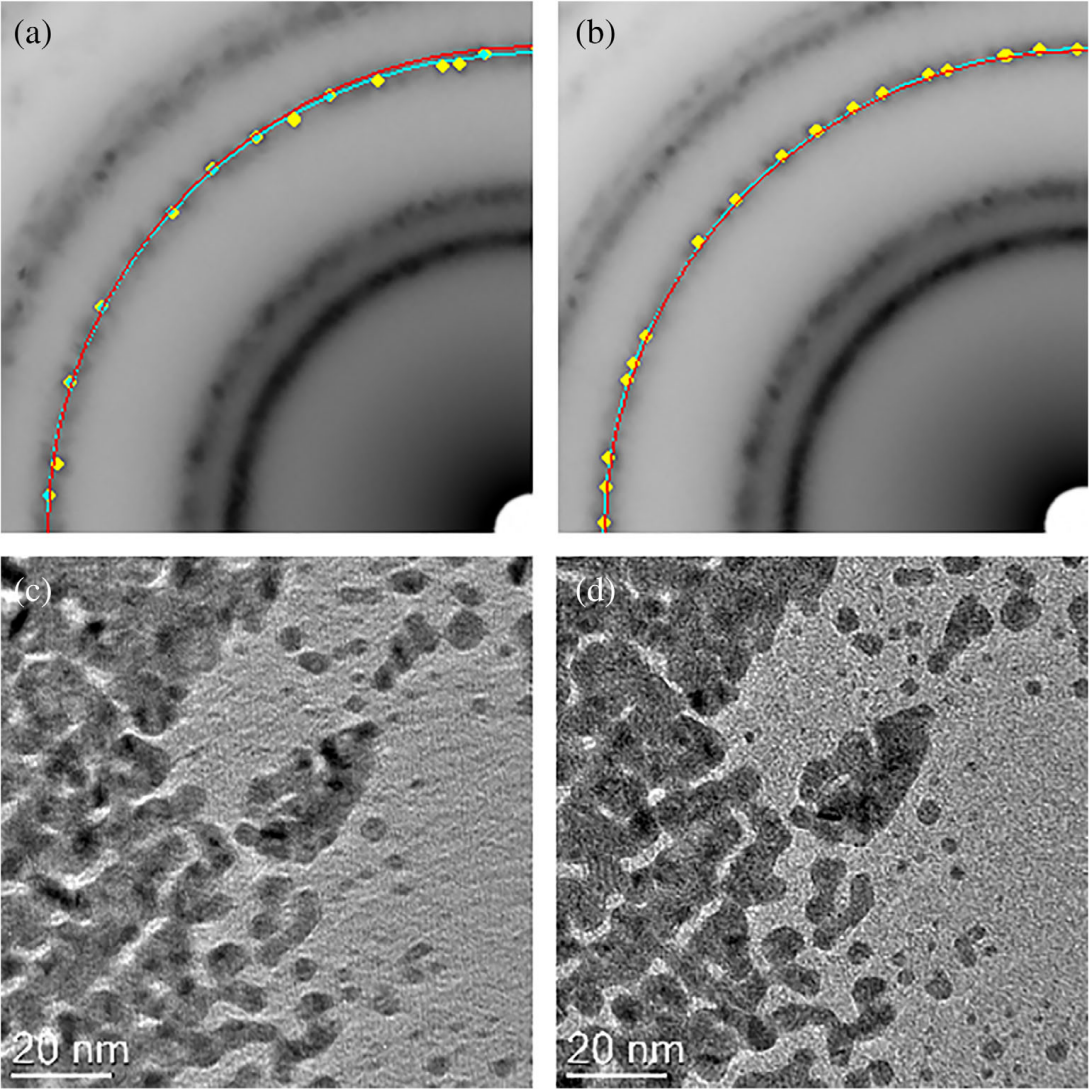
Application of the SADP Distortion Corrector script: (a) with the objective stigmators optimized for imaging a distorted diffraction pattern is obtained (yellow points/cyan line is the ellipse fitted to the diffraction ring and the red line is the equivalent circle of radius equal to the major axis of the ellipse); (b) after applying the saved optimum objective stigmator values for diffraction a distortion‐free pattern is obtained and the best‐fit ellipse and equivalent circle are in much closer registry; (c) switching from diffraction to imaging mode with optimum objective stigmator values for diffraction in place results in a highly astigmatic image; (d) recall of the saved optimum objective stigmator settings from for imaging results in a astigmatism‐free image

Figure 3 illustrates the application of this method. Following column alignment in imaging mode and switching to diffraction mode, a distorted diffraction pattern (1.6% distortion) is present (Figure 3a). The best‐fit ellipse (cyan line) fitted to a diffraction ring and the equivalent circle (red line) of radius equal to the major axis of the ellipse clearly deviate at the top right of the figure, due to ellipticity of the diffraction ring. After applying the objective stigmator values optimized for diffraction stored in the script, the diffraction pattern distortion is reduced to 0.3% and the best‐fit ellipse and equivalent circle are now in much closer registry (Figure 3b). After completing the diffraction work and switching back to imaging mode, the objective stigmator settings (optimized for diffraction) result in a severely astigmatic image (Figure 3c). Restoration of the optimum stigmator settings for imaging from the script, restores astigmatism‐free imaging (Figure 3d).

The distortion of a diffraction pattern in diffraction mode is relatively insensitive to the objective stigmator values (compared to image distortion in imaging mode) and the optimum objective stigmator values for distortion‐free diffraction are likely to be quite stable and to not require recalibration, provided no major realignments or column maintenance are carried out. In order to obtain reliable and consistent electron diffraction data, it is essential to standardize the diffraction conditions used (Mitchell, 2008a). Lens degaussing (twice) upon switching from imaging to diffraction modes is strongly advised, to ensure consistent camera length calibration and distortion correction. The scripts reported in this work enable accurate determination and application of the optimum objective stigmator values required to produce distortion‐free diffraction patterns in a facile manner. This technique has been used to demonstrate a better than five‐fold reduction in distortion, effectively eliminating distortion in electron diffraction.

## 4 CONCLUSIONS

Diffraction patterns acquired on a TEM contain distortions arising from column defects. A script‐based method of measuring distortion and correcting it using the objective lens stigmators has been developed and applied. A second script has been developed which allows the optimum objective lens stigmator values to be effortlessly applied in their respective imaging and diffraction modes by a click of a button. Using this method, pattern distortion has been reduced five‐fold from 1.6% to 0.3%. Since the precision in measurement is of the same order, this method effectively results in distortion‐free electron diffraction.

## CONFLICT OF INTEREST

The author declares no potential conflict of interest.

## Data Availability

Scripts used in this script are available for free download from the author's website (www.dmscripting.com). Data used in this publication are available from the author by request.

## References

[jemt24124-bib-0001] Capitani, G. C. , Oleynikov, P. , Hovmöller, S. , & Mellini, M. (2006). A practical method to detect and correct for lens distortion in the TEM. Ultramicroscopy, 106, 66–74. 16046067 10.1016/j.ultramic.2005.06.003

[jemt24124-bib-0002] Hou, V. D.‐H. , & Li, D. (2008). A method to characterize and correct elliptical distortion in electron diffraction patterns. Microscopy Today, 16, 36–41.

[jemt24124-bib-0003] Mitchell, D. R. G. (2008a). DiffTools: Electron diffraction software tools for DigitalMicrograph™. Microscopy Research and Technique, 71, 588–593. 18567012 10.1002/jemt.20591

[jemt24124-bib-0004] Mitchell, D. R. G. (2008b). Circular Hough transform diffraction analysis: A software tool for automated measurement of selected area electron diffraction patterns within DigitalMicrograph™. Ultramicroscopy, 108, 363–374. 10.1016/j.ultramic.2007.06.003 17643819

[jemt24124-bib-0005] Mitchell, D. R. G. , & Schaffer, B. (2005). Scripting‐customised microscopy tools for DigitalMicrograph™. Ultramicroscopy, 103, 319–332. 15885435 10.1016/j.ultramic.2005.02.003

[jemt24124-bib-0006] Mitchell, D. R. G. , & Van den Berg, J. A. (2016). Development of an ellipse fitting method with which to analyse selected area electron diffraction patterns. Ultramicroscopy, 160, 140–145. 26495808 10.1016/j.ultramic.2015.10.009

[jemt24124-bib-0007] Mugnaioli, E. , Capitani, G. C. , Nieto, F. , & Mellini, M. (2009). Accurate and precise lattice parameters by selected‐area electron diffraction in the transmission electron microscope. American Mineralogist, 94, 793–800.

[jemt24124-bib-0008] Xiong, Z. , Mitchell, D. R. G. , Saleh, A. A. , & Pereloma, E. V. (2018). Tetragonality of bcc phases in the transformation‐induced plasticity steel. Metallurgical and Materials Transaction A, 4, 5925–5929.

